# Deconstructing the Direct Reciprocal Hippocampal-Anterior Thalamic Pathways for Spatial Learning

**DOI:** 10.1523/JNEUROSCI.0874-20.2020

**Published:** 2020-09-02

**Authors:** Andrew J.D. Nelson, Lisa Kinnavane, Eman Amin, Shane M. O'Mara, John P. Aggleton

**Affiliations:** ^1^School of Psychology, Cardiff University, Wales, CF10 3AT, United Kingdom; ^2^Institute of Neuroscience, Trinity College Dublin 2, Dublin, Ireland

**Keywords:** amnesia, anterior thalamic nuclei, DREADDs, hippocampus, spatial learning, subiculum

## Abstract

The hippocampus is essential for normal memory but does not act in isolation. The anterior thalamic nuclei may represent one vital partner. Using DREADDs, the behavioral consequences of transiently disrupting anterior thalamic function were examined, followed by inactivation of the dorsal subiculum. Next, the anterograde transport of an adeno-associated virus expressing DREADDs was paired with localized intracerebral infusions of a ligand to target specific input pathways. In this way, the direct projections from the anterior thalamic nuclei to the dorsal hippocampal formation were inhibited, followed by separate inhibition of the dorsal subiculum projections to the anterior thalamic nuclei. To assay spatial working memory, all animals performed a reinforced T-maze alternation task, then a more challenging version that nullifies intramaze cues. Across all four experiments, deficits emerged on the spatial alternation task that precluded the use of intramaze cues. Inhibiting dorsal subiculum projections to the anterior thalamic nuclei produced the severest spatial working memory deficit. This deficit revealed the key contribution of dorsal subiculum projections to the anteromedial and anteroventral thalamic nuclei for the processing of allocentric information, projections not associated with head-direction information. The overall pattern of results provides consistent causal evidence of the two-way functional significance of direct hippocampal-anterior thalamic interactions for spatial processing. At the same time, these findings are consistent with hypotheses that these same, reciprocal interactions underlie the common core symptoms of temporal lobe and diencephalic anterograde amnesia.

**SIGNIFICANCE STATEMENT** It has long been conjectured that the anterior thalamic nuclei might be key partners with the hippocampal formation and that, respectively, they are principally responsible for diencephalic and temporal lobe amnesia. However, direct causal evidence for this functional relationship is lacking. Here, we examined the behavioral consequences of transiently silencing the direct reciprocal interconnections between these two brain regions on tests of spatial learning. Disrupting information flow from the hippocampal formation to the anterior thalamic nuclei and vice versa impaired performance on tests of spatial learning. By revealing the conjoint importance of hippocampal-anterior thalamic pathways, these findings help explain why pathology in either the medial diencephalon or the medial temporal lobes can result in profound anterograde amnesic syndromes.

## Introduction

**Figure 1.**
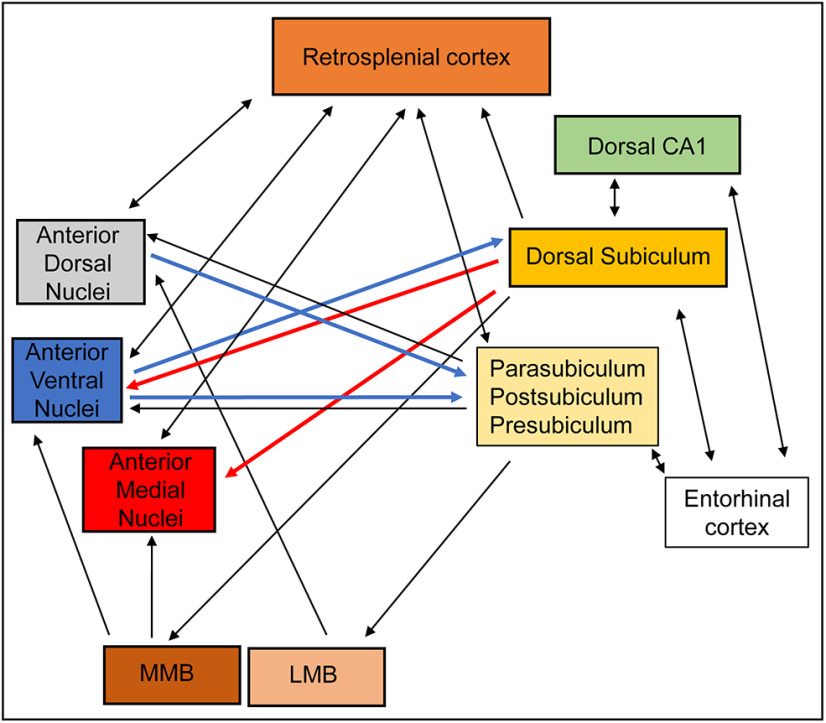
Hippocampal-diencephalic-retrosplenial connectivity. Schematic showing the pattern of connectivity between the hippocampal formation (and parahippocampal cortices) with the medial diencephalon (ATN and mammillary bodies) and retrosplenial cortex. Arrows indicate the direction of the principal connections within this network. Double-ended arrows indicate reciprocal connections. Blue arrows indicate the connections targeted in Experiment 3. Red arrows indicate connections targeted in Experiment 4. LMB, Lateral mammillary bodies; MMB, medial mammillary bodies.

The importance of the hippocampal formation for memory remains irrefutable, yet the structure does not act in isolation. Among potential critical partners, the anterior thalamic nuclei (ATN) have a strong claim. Initial evidence comes from the dense, reciprocal connections between the hippocampal formation and these nuclei (Meibach and Siegel, 1977; Shibata, 1993a; Wright et al., 2010; Christiansen et al., 2016). While the anteromedial and anteroventral thalamic nuclei are principally interconnected with the dorsal subiculum, the anterodorsal thalamic nucleus is largely interconnected with parahippocampal areas, including the postsubiculum and presubiculum (Meibach and Siegel, 1977; van Groen and Wyss, 1990a,b; Shibata, 1993a; Christiansen et al., 2016) (Fig. 1). [Throughout, the terms hippocampal formation and hippocampal refer to the dentate gyrus, the CA fields, and the subiculum (Burwell and Witter, 2002)].

Further relevant evidence comes from neuropsychological studies showing that pathology or disconnection of the human ATN appears closely associated with anterograde amnesia (Harding et al., 2000; Van der Werf et al., 2003; Carlesimo et al., 2011; Segobin et al., 2019). While mediodorsal thalamic damage has also been implicated in aspects of diencephalic amnesia, its functions are more closely aligned with those of PFC (Mitchell, 2015; Wolff et al., 2015). Moreover, ATN lesions in rodents produce profound deficits on spatial tasks that are hippocampal-dependent, deficits that are arguably more severe than for any other single-brain region outside the hippocampus (Aggleton and Nelson, 2015; Perry et al., 2018). Nevertheless, current support for a reciprocal hippocampal–anterior thalamic mnemonic system remains reliant on indirect evidence.

One approach to testing hippocampal–anterior thalamic interactions more directly has involved the use of surgical disconnections (e.g., a unilateral hippocampal lesion paired with a unilateral anterior thalamic lesion in the opposite hemisphere). In rats, this procedure impairs both reference and working memory tests of spatial learning (Warburton et al., 2000, 2001; Henry et al., 2004). Unfortunately, such disconnections fail to reveal the anatomic direction of any behavioral effects, nor can contributions from indirect disconnections be excluded. The presence of many crossed hippocampal projections to the anterior thalamus (Mathiasen et al., 2019) adds further complications. Furthermore, permanent lesions in both sites result in retrosplenial cortex dysfunctions (Albasser et al., 2007; Garden et al., 2009) that could contribute to any observed behavioral deficits. These multiple limitations can, however, be obviated by combining the anterograde transport of a chemogenetic construct from a predetermined starting site (e.g., hippocampal formation) to a target (e.g., ATN) with the intracerebral infusion of a ligand to activate the construct selectively at that target site (Roth, 2016; Smith et al., 2016).

In a two-phase design, inhibitory designer receptor exclusively activated by designer drugs (DREADDs) activated by systemic injection of a ligand were first used to test the functional consequences of transiently inactivating the ATN (Experiment 1) and then the dorsal subiculum (Experiment 2). In Phase 2, the direct connections between these same sites were isolated by combining the axonal transport of DREADDs with localized intracerebral injections of the ligand, clozapine, to target their respective efferent terminations (Smith et al., 2016; Gomez et al., 2017; Campbell and Marchant, 2018). By these means, we first examined the effects of transiently inactivating the anterodorsal and anteroventral terminations within the subiculum and parahippocampal area (Experiment 3). Last, we sought to inhibit the dorsal subiculum efferents within the anteroventral and anteromedial thalamic nuclei (Experiment 4). Throughout, animals were tested on reinforced T-maze alternation, a measure of spatial working memory that is acutely sensitive to both hippocampal and anterior thalamic damage (Aggleton et al., 1986, 1996; McHugh et al., 2008; Alcaraz et al., 2016).

## Materials and Methods

### Subjects

All experiments involved experimentally naive, male Lister Hooded rats (Envigo). The rats were housed in pairs in a temperature-controlled room, with Lignocel bedding (#03018200115, IPS). At the time of surgery, the rats in Experiment 1 weighed 269-304 g, those in Experiment 2 weighed 309-464 g, those in Experiment 3 weighed 313-358 g, and those in Experiment 4 weighed 287-347 g. Lighting was kept on a 12 h light/dark cycle, light from 0800-2000. Water was available *ad libitum* throughout the experiments. For all behavioral experiments, the animals were placed on a food-restricted diet where they were able to gain weight. Their weights did not fall below 85% of their free-feeding weights. All experiments were conducted in accordance with UK Animals (Scientific Procedures) Act, 1986 and EU directive (2010/63/EU).

In Experiment 1, animals received injections of either inhibitory DREADDs (ATN_iDRD; *n* = 13 animals) or a control virus (ATN_Control; *n* = 9) into the ATN. In Experiment 2, animals received injections of either inhibitory DREADDs (DSub_iDRD; *n* = 12 animals) or a control virus (DSub_Control; *n* = 6) into dorsal subiculum. Experiment 3 involved injections of either inhibitory DREADDs (ATN→Sub_iDRD; *n* = 18 animals) or a control virus (ATN→Sub _Control; *n* = 11) into the ATN combined with cannulae targeting efferents in the caudal subiculum/parahippocampal region. Given the diffuse nature of the anterior thalamic terminations within the subiculum and parahippocampal area, a higher number of animals were used in Experiment 3 to achieve sufficient power. Finally, Experiment 4 involved injections of either inhibitory DREADDs (DSub→ATN_iDRD; *n* = 7 animals) or a control virus (DSub→ATN _Control; *n* = 5) into the dorsal subiculum combined with cannulae targeting efferents in the ATN.

### Surgical procedures

All rats were anesthetized with isoflurane (4% induction, 2% thereafter). Next, each rat was placed in a stereotaxic frame (David Kopf Instruments), with the incisor bar set at 5.0 mm to the horizontal plane, except for the implantation of cannulae into the subiculum (Experiment 4) for which the skull was flat. For analgesic purposes, lidocaine was administered topically to the scalp (0.1 ml of 20 mg/ml solution; B. Braun) and meloxicam was given subcutaneously (0.06 ml of 5 mg/ml solution, Boehringer Ingelheim). A craniotomy was then made directly above the target region and the dura cut to expose the cortex.

#### Stereotaxic injections of inhibitory DREADDs (Experiments 1-4)

In all experiments, the animals received either injections of the inhibitory DREADD (experimental groups) AAV5-CaMKIIa-hM4Di(Gi)-mCherry (Addgene) or (control groups) AAV5-CaMKIIa-EGFP (Addgene). Group allocation was random.

Preliminary data demonstrated that this DREADD construct, when injected into the ATN and activated by systemic clozapine (4 mg/kg), led to reductions in Fos-positive cells in granular retrosplenial cortex (−34.3%), dysgranular retrosplenial cortex (−31.2%), and dorsal subiculum (−44.4%) but only minor changes in the CA1 (−14.1%) and CA3 (−1.45%) subfields of the dorsal hippocampus.

Injections were made via a 10 μl Hamilton syringe attached to a moveable arm mounted to the stereotaxic frame. The injections were controlled by a microsyringe pump (World Precision Instruments) set to a flow rate of 0.1 μl/min, and the needle left *in situ* for a further 5 min to allow for diffusion of the bolus. The injection coordinates (relative to bregma), volumes, and virus titer for each experiment were as follows:

*Experiment 1*: AAV5-CaMKIIa-hM4Di(Gi)-mCherry (titer 4.4 × 10^12^ GC/ml) or AAV5-CaMKIIa-EGFP (titer 4.2 × 10^12^ GC/ml) was injected into the following sites in the ATN: AP −0.1, ML ±0.8, DV −6.8 (0.65 µl); AP −0.2, ML ±1.5, DV −6.2 (0.8 µl).

*Experiment 2*: AAV5-CaMKIIa-hM4Di(Gi)-mCherry (titer 4.4 × 10^12^ GC/ml) or AAV5-CaMKIIa-EGFP (titer 4.2 × 10^12^ GC/ml) was injected into the following site in the dorsal subiculum: AP −4.8, ML ±3.1, DV −5.3 (0.6 µl).

*Experiment 3*: AAV5-CaMKIIa-hM4Di(Gi)-mCherry (titer 4.3 × 10^12^ GC/ml) or AAV5-CaMKIIa-EGFP (titer 4.3 × 10^12^ GC/ml) was injected into the following sites in the ATN: AP −0.1, ML ±0.8, DV −6.8 (0.4 µl); AP −0.2, ML ±1.5, DV −6.2 (0.65 µl).

*Experiment 4*: AAV5-CaMKIIa-hM4Di(Gi)-mCherry (titer 2.6 × 10^13^ GC/ml) or AAV5-CaMKIIa-EGFP (titer 4.3 × 10^12^ GC/ml) was injected into the following sites in the dorsal subiculum: AP −4.8, ML ±3.0, DV −5.1 (0.6 µl); AP −5.8, ML ±3.3, DV-5.3 (0.4 µl).

#### Stereotaxic implantation of guide cannulae (Experiments 3 and 4)

In Experiment 3, to target anterior thalamic projections to the subiculum and parahippocampal region (Fig. 1), a craniotomy was drilled in each hemisphere and a single cannula (Plastics One) was implanted per hemisphere in the caudal subiculum region (26 gauge cut to length of 6 mm) at the following coordinates from bregma (flat skull): AP −7.0, ML ±3.5, DV −3.5 (from dura). The choice of cannulae location was based on the known termination of anteroventral and anterodorsal efferents in the postsubiculum, parasubiculum, and dorsal subiculum (van Groen and Wyss, 1990a,b; Shibata, 1993a).

To target subicular efferents to the ATN (Experiment 4) (Fig. 1), a craniotomy was drilled in each hemisphere, and bilateral guide cannulae (Plastics One) were implanted (26 gauge, cut to a length of 4.8 mm, center to center distance of 2.7 mm) in the ATN at the following coordinates from bregma: AP −0.1, ML ±1.35, DV −4.4 (from dura). Again, cannulae placement was based on the known termination of dorsal subicular efferents within the anteromedial and anteroventral thalamic nuclei (Wright et al., 2010, 2013; Christiansen et al., 2016).

Cannulae were held in place by bone cement (Zimmer Biomet) and anchored to the skull with four fixing screws (Precision Technology Supplies). Removable obturators (Plastic One) were inserted into the guide cannulae to prevent the cannulae from blocking.

For all animals (Experiments 1-4), the surgical site was closed using sutures, and the analgesic bupivacaine (Pfizer) was injected between the suture sites. A topical antibiotic powder Clindamycin (Pfizer) was then applied to the site. Animals were administered a subcutaneous injection of glucose-saline (5 ml) for fluid replacement before being placed in a recovery chamber until they regained consciousness. Animals were monitored carefully postoperatively with food available *ad libitum* until they had fully recovered, with behavioral pretraining commencing ∼7 d after surgery.

### Behavior

#### Apparatus

**Figure 2.**
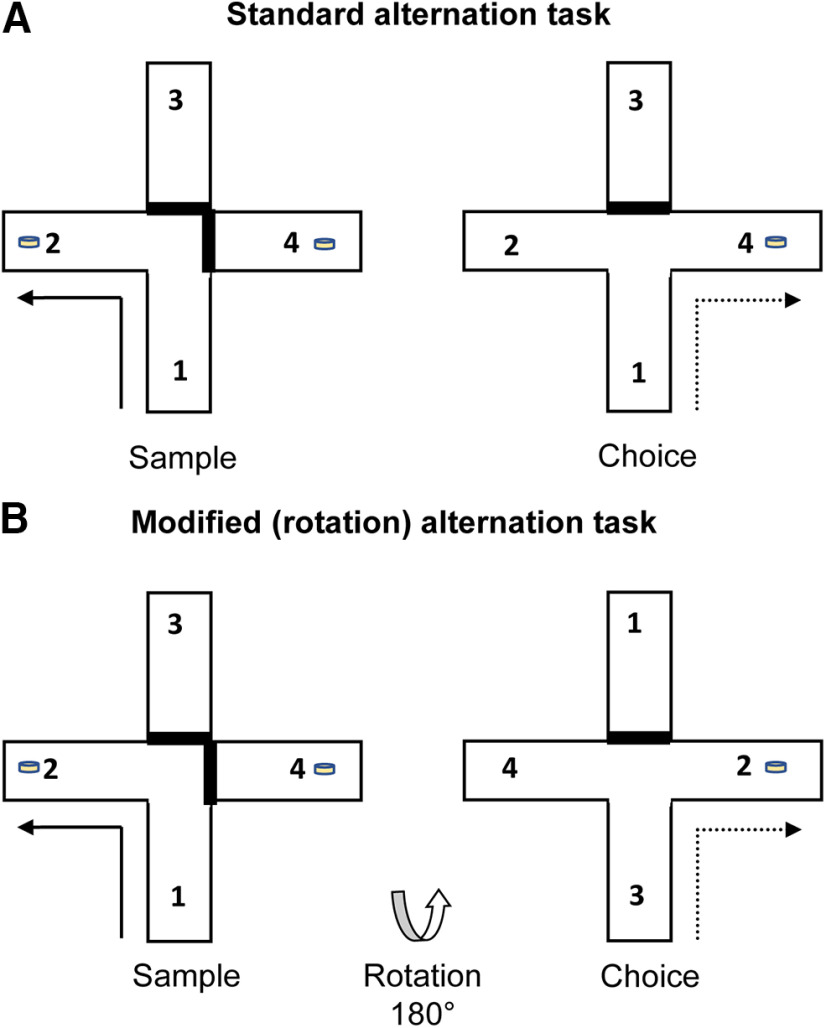
T-maze spatial alternation task. Schematic of the test protocols for the forced-choice alternation task run in a single maze. Solid lines indicate the sample phase. Dashed lines indicate the correct response in the choice phase. Food rewards were located in wells at the end of the arms. ***A***, Standard trials permitted the use of multiple cue types. ***B***, The task was modified to nullify the value of intramaze cues by rotating the apparatus either 90° or 180° between the sample phase and choice phase. The numbers indicate the arm identity. ***B***, Illustration of a trial in which the maze was rotated 180° between the sample phase and choice phase.

Testing was conducted in a modifiable four-arm (cross-shaped) maze (Fig. 2). The four arms (70 cm long, 10 cm wide) were made of wood while the walls (17 cm high) were made of clear Perspex. At any time, one of the arms could be blocked off to form a T-shaped maze. Aluminum barriers could be positioned ∼25 cm from the end of each arm to create a start area. For all experiments, the location of the start arm remained constant such that the T-maze was in the same orientation throughout testing (Fig. 2). The maze, which was supported by two stands (94 cm high), was situated in a rectangular room (280 cm × 280 cm × 210 cm) with salient visual cues located on the walls.

#### Procedure

Behavioral training consisted of three stages: habituation, training on the standard T-maze task (both before and after surgery), and training on the modified version (with rotation). Test sessions in which DREADDs were activated followed. To allow for optimal viral expression, the critical test sessions were all conducted at least 3 weeks after surgery. Behavioral testing was conducted by an experimenter blind to group allocation.

##### Pretraining

Pretraining began with 4 d of habituation to the maze. On day 1, Cheerios halves (Nestle) were scattered down each of the arms and two halves in each food well, and the rats allowed to explore the maze for 5 min. On day 1 only, rats were habituated in cage pairs. On days 2 and 3, the number of Cheerios was increased to four halves in each well and the rats were habituated to the maze for 5 min. On day 4, the number of Cheerios was reduced to two halves in each well and the aluminum barrier was placed at the entrance of one of the arms. The rats were again allowed to explore the maze for 5 min. During each habituation session, the reward pellets were continuously replaced so that no arm was found to be empty on return

##### Standard T-maze task (all cue types available)

Throughout, the rats received eight trials per daily session. Each trial consisted of a forced “sample” run followed by a “choice” run. Although all testing was conducted in a cross-maze, it was modified by placing a barrier blocking access to the arm directly in line with the start arm (effectively turning it into a T-maze configuration) (Fig. 2*A*).

During the forced sample run, one of the side arms of the maze was blocked by an aluminum barrier. After the rat turned into the preselected arm, it ate the reward (half a Cheerio) that had been previously placed in the food well. The rat was then picked up from the maze and immediately returned to the start arm; ∼10 s after the end of the sample phase, the choice phase began. The rat was allowed to run up the stem of the maze and was now given a free choice between the left and the right turn arms. The rat received a food reward only if it selected the goal arm opposite to that in the sample run (i.e., nonmatching) (Fig. 2). The choice of the sample arm (left or right) was randomly assigned with the only stipulation that no arm could be selected as the sample on more than two consecutive trials.

At the start of each session, rats were taken from the holding room to an adjacent experimental room. Rat were then tested individually so that the intertrial interval was ∼20 s.

The rats were trained for a minimum of 12 d on the standard version of the T-maze. Thereafter, each rat underwent two test sessions on the standard T-maze task. In one of these test sessions, the animals received injections or infusions of the DREADD ligand clozapine (see below for details). For the other test session, the animals received a control saline injection or infusion. The test sessions proceeded as during the training (i.e., each rat received 8 trials on the standard task per test session). The order of testing (i.e., DREADD activation or control injection/infusion) was counterbalanced across animals.

##### Modified T-maze task with rotation

The modified version of the T-maze task nullifies the value of intramaze cues. To this end, the maze was rotated between the sample and choice phases (Fig. 2*B*). Once the sample phase was completed, the rat was removed from the sample arm and the start arm was rotated 90° (half the trials) or 180° (the remaining trials). The rat was put back in the new “south” arm. In order to prevent the rats from acquiring a rule, the direction and magnitude of the rotation (either clockwise or counterclockwise, 90° or 180°) were randomly selected so that on half the trials it was rotated clockwise and on the other half it was rotated counterclockwise (there were never more than two successive rotations in the same direction or the same magnitude) (Fig. 2*B*). Training on the modified version commenced after the test sessions on the standard task. The rats were trained on the modified version of the task for a minimum of 4 d before testing. There were two test sessions on the modified version of the task. Before each of these sessions, the animals received injections or infusions of the DREADD ligand clozapine or saline. The order of testing (i.e., DREADD ligand or control injection/infusion) was counterbalanced across animals.

##### Systemic injection of clozapine (Experiments 1 and 2)

Clozapine was used to activate DREADDs (Gomez et al., 2017; Tan et al., 2020). Although there are concerns (Ilg et al., 2018) about potential off-target effects of this compound, Ilg et al. (2018) found no evidence thatlow-dose clozapine affected working memory. Pilot data showed that clozapine effectively activated the hm4Di DREADD receptor without apparent concomitant nonspecific effects. Our preliminary data found no differences by group in the mean time to complete the sample (max *F*_(1,11)_ = 0.72, *p* = 0.42) or choice stages (max *F*_(1,11)_ = 1.33, *p* = 0.27) of the T-maze task. More importantly, to control for any potential off-target effects, all test sessions involved comparisons between control and DREADD groups both administered with clozapine. Furthermore, the standard and modified versions of the T-maze task are matched in terms of the sensorimotor and motivational requirements.

Clozapine dihydrochloride (Hello Bio) as salt was dissolved in sterile saline to an injection volume of 1 ml/kg. In Experiment 1, rats were administered a dose of 4 mg/kg (3.27 mg/kg freebase). For Experiment 2, the dose was lowered to 2 mg/kg (1.64 mg/kg freebase), as preliminary work demonstrated that this lower dose effectively activated DREADDs expressed in the dorsal subiculum.

Both DREADD groups and control groups received an intraperitoneal injection of clozapine 30 min before testing. For the alternate test session, rats received an intraperitoneal injection (1 ml/kg of 0.9% saline), again 30 min before testing.

##### Intracranial infusion of clozapine (Experiments 3 and 4)

Clozapine dihydrochloride salt (Hello Bio) was dissolved in sterile saline at a dose of 1 µg/µl (0.82 µg/µl, freebase). Rats were lightly restrained, the obturators removed, and 33-gauge stainless-steel infusion cannulae (Plastic One) that projected 0.5 mm beyond the tip of the guide cannulae were inserted bilaterally into the subiculum (Experiment 3) or ATN (Experiment 4). Each pair of infusion cannula was connected to two 5 μl Hamilton syringes mounted on two infusion pumps (Harvard Apparatus). A volume of 1 μl per infusion site was infused over 1 min. The infusion cannulae were left *in situ* for a further 1 min to allow absorption of the bolus. The infusion cannulae were then removed and the obturators replaced. The animals were returned to their home cage. After 15 min, testing began. For the alternate (saline control) test session, rats underwent the same procedure, except 1 μl of sterile saline was infused.

### Histology

Animals were administered an intraperitoneal injection of a lethal dose of sodium pentobarbital (2 ml/kg, Euthatal, Marial Animal Health) and transcardially perfused with 0.1 m PBS, followed by 4% PFA in 0.1 M PBS. Brains were removed, postfixed in PFA for 2 h, and then placed in 25% sucrose solution for 24 h at room temperature on a stirring plate.

Brains were cut into 40 μm coronal sections using a freezing microtome (8000 sledge microtome, Bright Instruments), and a series of 1 in 4 sections was collected in PBS for fluorescence analysis. An additional series was collected for cresyl staining.

#### Immunohistochemistry for DREADD expression

Immunohistochemistry was conducted on the tissue to enhance the fluorescence signal of mCherry (DREADD groups) or EGFP (control groups). The first series of sections was transferred from PBS into a blocking solution of 5% NGS in PBS with Triton X-1000 (PBST) and incubated for 1 h. The sections were then transferred into the primary antibody solution of rabbit-anti-mCherry or chicken polyclonal anti-GFP (Abcam) at a dilution of 1:1000 in PBST with 1% NGS and incubated for 24 h. Sections were washed 4 times in PBST and transferred to a secondary antibody solution of goat-anti-rabbit (Dylight AlexaFlour-594, Vector Laboratories) or goat-anti-chicken (Invitrogen) at a dilution of 1:200 at PBST. From this point onward, the sections were kept in the dark. Sections were incubated for 1 h before being washed 3 times in PBS. Sections were mounted onto gelatin-subbed glass slides and dried overnight before being immersed in xylene and coverslipped using DPX (Thermo Fisher Scientific). All incubations were on a stirring plate at room temperature, and all washes were for 10 min.

### Experimental design and statistical analysis

The behavioral data were analyzed by ANOVA with within-subject factors of injection or infusion (saline vs clozapine) and between-subject factor of group (DREADD vs control). Where appropriate, significant interactions were analyzed by simple main effects based on the pooled error term (Howell, 2010). One-sample *t* tests (two-tailed) assessed whether performance was above chance levels (i.e., 50%). Partial η squared (η_p_^2^) is given as a measure of effect size. The α level was set at *p* < 0.05.

As standard hypothesis testing does not assess whether the absence of a significant effect provides good evidence for no true relationship, we have supplemented standard hypothesis testing with Bayesian analysis where appropriate. Bayesian tests explicitly calculate the relative probabilities of the null or alternative hypothesis. The Bayes factor B_01_ denotes when the data support the null hypothesis. A Bayes factor between 1 and 3 gives anecdotal support, a factor between 3 and 10 suggests moderate evidence, and beyond 10 indicates strong evidence (Rouder et al., 2012). Statistical analysis was performed on JASP computer software (version 0.11.1) and SPSS (IBM Statistics, version 25).

## Results

### Experiment 1: ATN are required for spatial working memory

**Figure 3.**
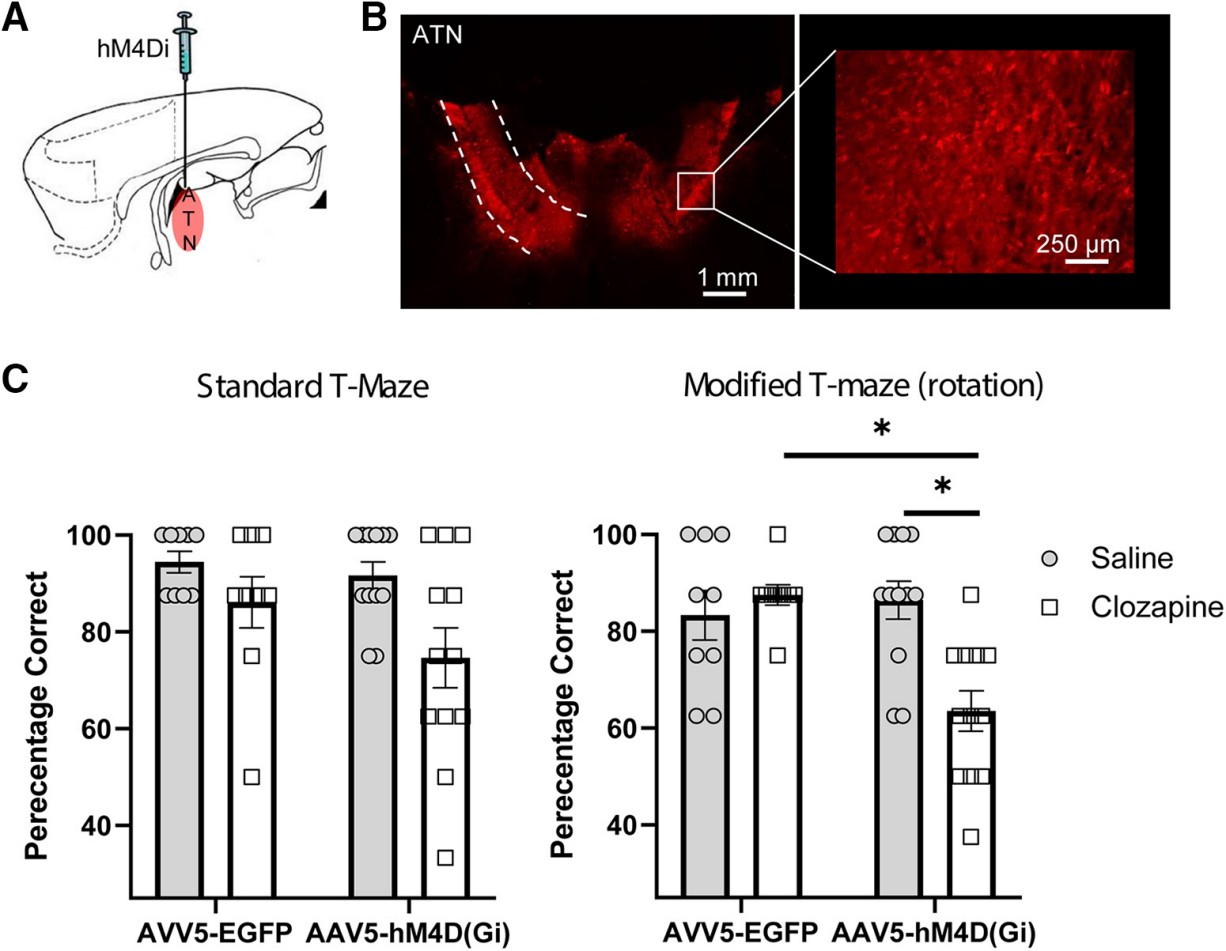
DREADD-mediated inhibition of ATN cortex impairs spatial working memory. ***A***, Sagittal schematic of rat forebrain. ***B***, AAV5-hM4D(Gi)-mCherry expression in ATN (coronal section). All animals displayed robust expression within the ATN and their efferent targets (see also Fig. 4). ***C***, DREADD activation did not reliably disrupt spatial working memory on the standard version of the alternation task, but performance was impaired on the modified version (maze rotation). The DREADD group was selectively impaired relative to their performance under saline (*p* < 0.001) as well as to the performance of controls under clozapine (*p* < 0.005). Plots represent mean percentage correct. Error bars indicate SEM. *denotes statistically significant differences.

**Figure 4.**
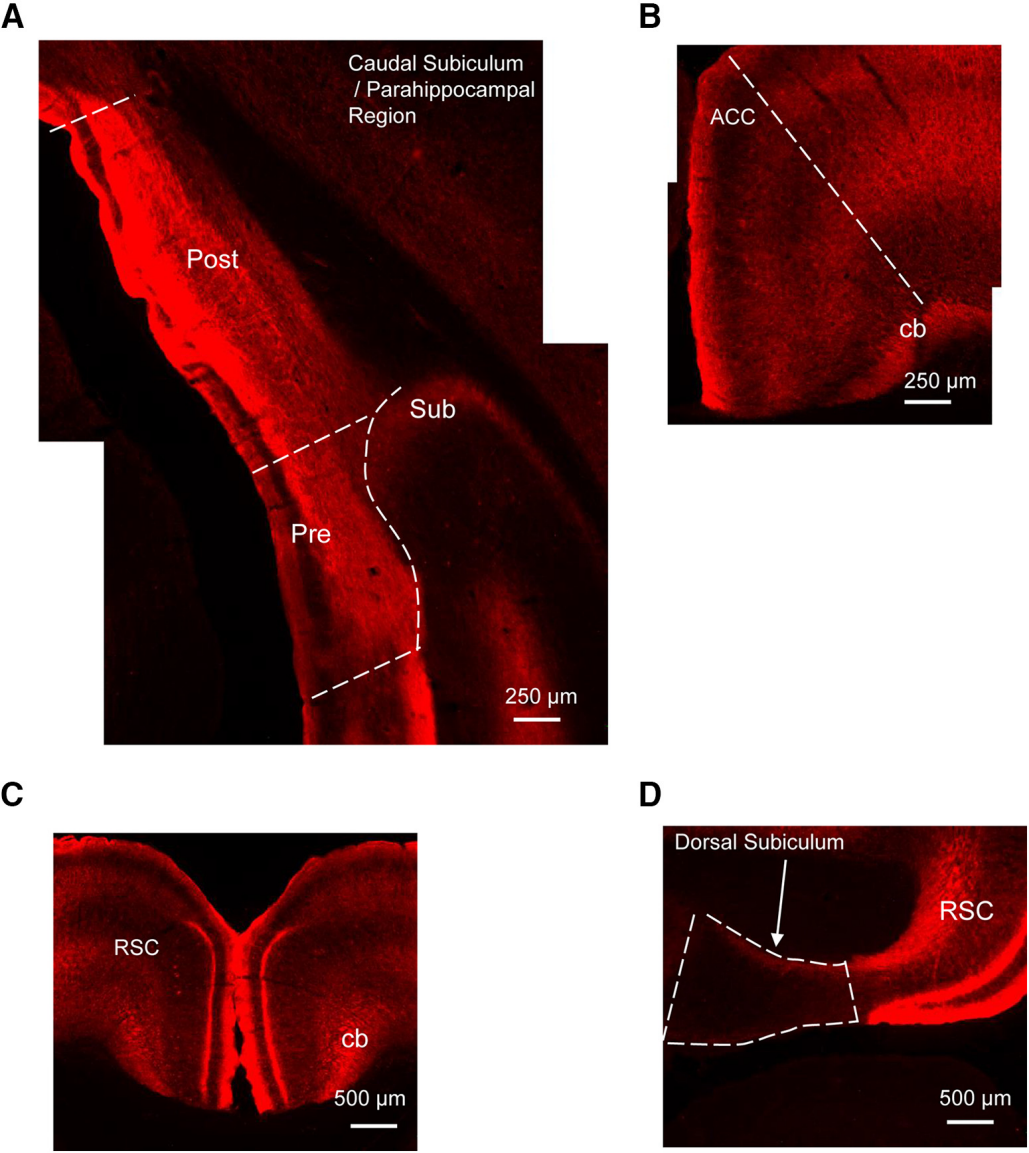
Pattern of transport of AAV5-hM4D(Gi)-mCherry following injections in the ATN (Experiments 1 and 3). ***A***, Anterograde transport within the caudal subiculum and parahippocampal region (right hemisphere). ***B***, Anterograde transport within the ACC (right hemisphere). ***C***, Anterograde transport within the retrosplenial cortex. ***D***, Absence of retrograde label in dorsal subiculum. All coronal sections. cb, cingulum bundle; Post, postsubiculum; Pre, presubiculum; RSC, retrosplenial cortex; Sub, subiculum.

To assay the role of ATN in spatially working memory, an adeno-associated virus carrying the hM4Di receptor (AAV5-CaMKIIa-hM4Di(Gi)-mCherry) was injected into the ATN (ANT_iDRD group) (Fig. 3*A*), while control animals received injections of the same virus (AAV5-CaMKlla-EGFP) not expressing the DREADD receptor (ATN_Control). Viral expression was assessed both in the ATN but also critically in efferent targets based on the known connectivity of the ATN (e.g., the retrosplenial cortex, ACC, caudal subiculum, and parahippocampal areas; see Figs. 1, 4). One case was excluded as there was little evidence of viral expression in the ATN. In the remaining animals, mCherry expression (ANT_iDRD group) and EGFP (ATN_Control) was evident throughout the ATN (Fig. 3*B*) as well as in its efferent targets (Fig. 4). Importantly, there was no evidence of retrograde label in sites with which the ATN are reciprocally connected (dorsal subiculum, retrosplenial cortex, ACC, parahippocampal sites) with the pattern of label in these sites (Figs. 1, 4*A-D*) consistent with anterograde transport as previously described with standard anatomic tracing methods (Shibata, 1993a,b). The final group numbers were ATN_iDRD = 12 and ATN_Control = 9.

There were no differences between the two groups during presurgical (*F*_(1,19)_ = 0.92, *p* = 0.35) or postsurgical training (*F*_(1,19)_ = 0.3, *p* = 0.59) on the standard T-maze alternation task (DREADDs not activated).

At the outset of the critical test sessions, clozapine was injected intraperitoneally to activate the DREADDs (Gomez et al., 2017). Figure 3*C* displays the mean performance of both groups following saline and clozapine injections before the standard T-maze task (all potential alternation cues available). Although performance in the ANT_iDRD group appeared to be attenuated following activation of the DREADDs, ANOVA yielded no statistical evidence that the two groups differed. ANOVA revealed a main effect of drug (*F*_(1,19)_ = 9.2, *p* = 0.007, η_p_^2^ = 0.325), but no interaction (*F*_(1,19)_ = 1.1, *p* = 0.3, η_p_^2^ = 0.054) or main effect of group (*F*_(1,19)_ 1.09 *p* = 0.18, η_p_^2^ = 0.095). However, a Bayesian ANOVA was also calculated, and the model based on the interaction revealed a B_01_ factor of 0.12, indicating no evidence in favor of the null hypothesis.

On the modified version of the task (maze rotated between the sample and choice stages of the procedure) (Fig. 2), there was a clear effect of DREADD activation on spatial alternation behavior (Fig. 3*C*). There was a main effect of drug (*F*_(1,19)_ = 5.0, *p* =0.037, η_p_^2^ = 0.209) and group (*F*_(1,19)_ = 4.9, *p* = 0.04, η_p_^2^ = 0.203) but also, critically, an interaction between these factors (*F*_(1,19)_ = 11.3, *p* = 0.003, η_p_^2^ = 0.372). While there was no effect of clozapine in the ATN_Control animals (*F*_(1,19)_ = 0.4, *p* = 0.55), the ANT_iDRD group was selectively impaired when the DREADDs were activated relative to their performance under saline (*F*_(1,19)_ = 25.0, *p* = 0.00008) as well as to the performance of the control rats under clozapine (*F*_(1,19)_ = 11.7, *p* = 0.003). Despite this impairment, performance in the ATN_iDRD group remained above chance levels (*t*_(11)_ = 3.0, *p* = 0.012). Subsequent focused analysis comparing the ATN-iDRD group (DREADDs activated) across the two task variants confirmed that there was no statistical evidence that performance differed by task (*F*_(1,11)_ = 2.02, *p* = 0.18, η_p_^2^ = 0.16).

### Experiment 2: dorsal subiculum is required for spatial working memory

**Figure 5.**
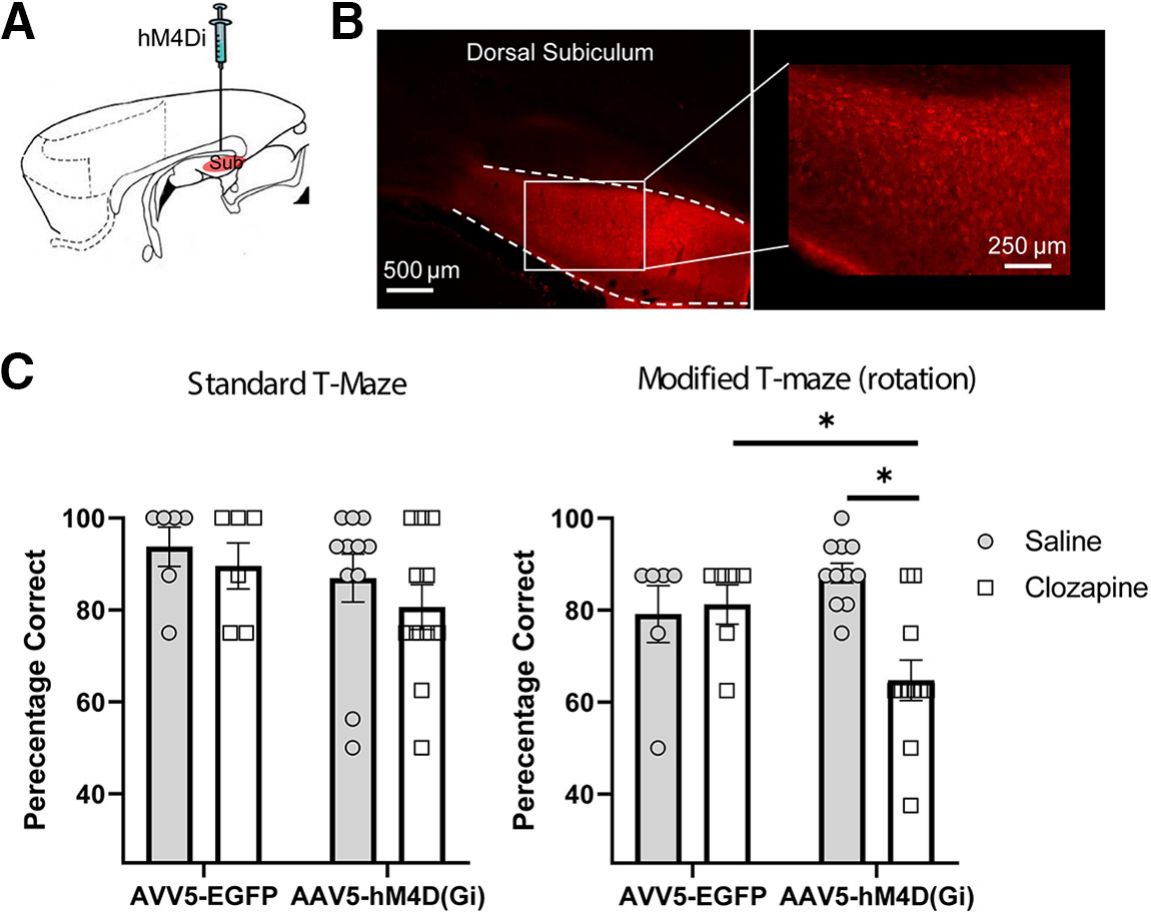
DREADD-mediated inhibition of dorsal subiculum impairs spatial working memory. ***A***, Sagittal schematic of rat forebrain. ***B***, AAV5-hM4D(Gi)-mCherry expression in dorsal subiculum (coronal section, right hemisphere). All animals displayed robust expression within the dorsal subiculum and their efferent targets (see also Fig. 6). ***C***, DREADD activation did not disrupt spatial working memory on the standard version of the alternation task, but performance was impaired on the modified version (maze rotation). The DREADD group was selectively impaired relative to their performance under saline (*p* < 0.001) as well as to the performance of controls under clozapine (*p* < 0.05). Plots represent mean percentage correct. Error bars indicate SEM. *denotes statistically significant differences.

**Figure 6.**
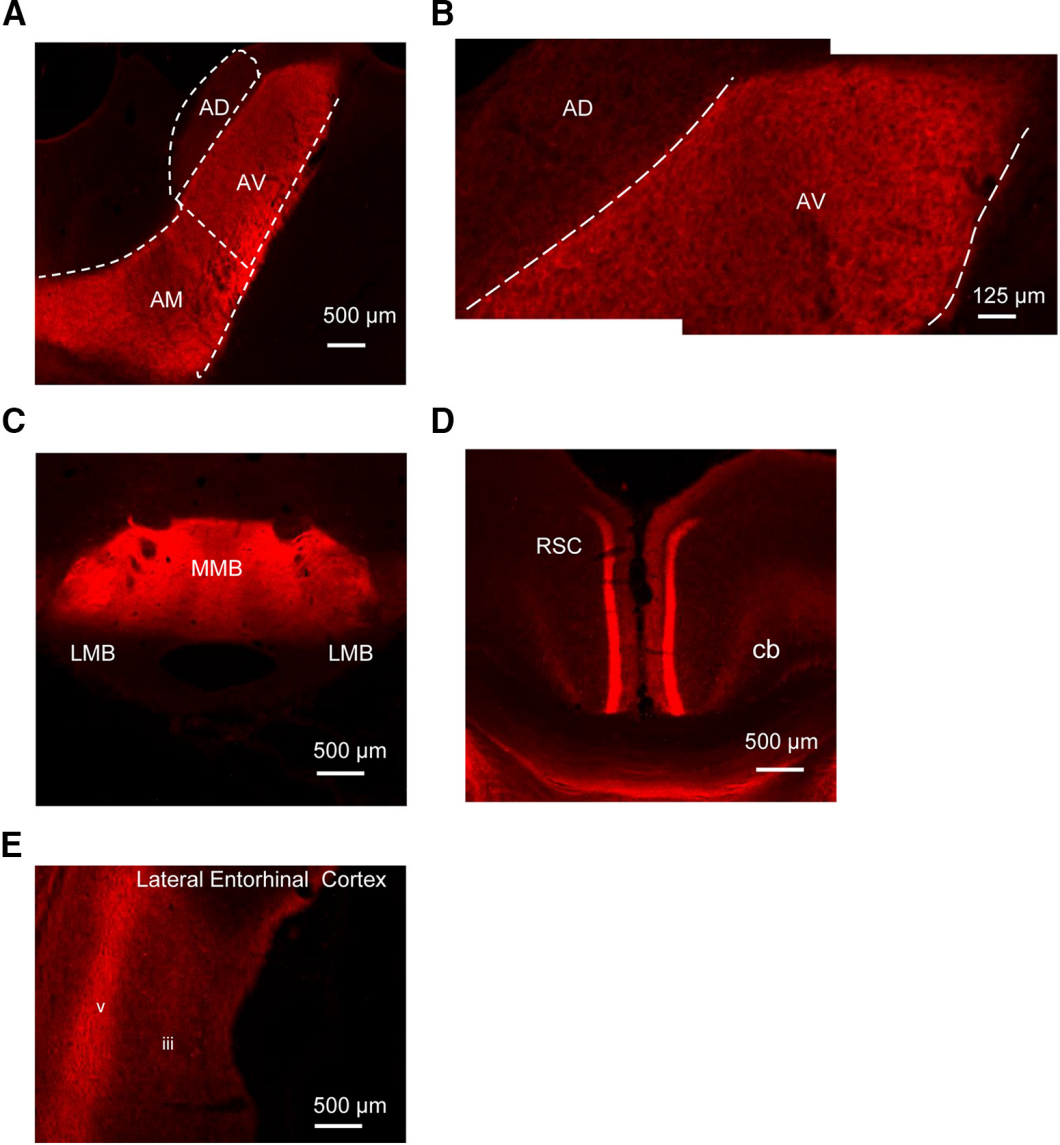
Pattern of transport of AAV5-hM4D(Gi)-mCherry following injections in the dorsal subiculum (Experiments 2 and 4). ***A***, Anterograde transport within the anteroventral and anteromedial thalamic nuclei (right hemisphere). ***B***, Contrast between pattern of anterograde transport in anteroventral and anterodorsal thalamic nuclei (right hemisphere). ***C***, Anterograde transport restricted to the medial mammillary bodies. ***D***, Anterograde transport within layers 2/3 of granular retrosplenial cortex. ***E***, Anterograde transport in deep layers of the lateral entorhinal cortex, absence of retrograde label in superficial layers. All coronal sections. AD, anterodorsal nucleus; AM, anteromedial nucleus; AV, anteroventral nucleus; cb, cingulum bundle; MMB, medial mammillary bodies; LMB, lateral mammillary bodies, RSC, retrosplenial cortex.

Next, the effects of activating DREADDs in the dorsal subiculum were examined on the same tests of spatial working memory (Fig. 5*A*). There was robust expression of mCherry within the dorsal subiculum in the experimental group (DorSub_IDRDs) and EGFP in the control animals (DorSub_Control) (Fig. 5*B*), but also anterograde label in known dorsal subicular efferents (Figs. 2, 6). Critically, the pattern of anterograde label was consistent with the known connectivity of the dorsal subiculum. For example, anterograde label was present in the anteroventral and anteromedial, but not the anterodorsal thalamic nuclei (Fig. 6*A*,*B*) (Christiansen et al., 2016). Similarly, anterograde label was largely restricted to layers 2 and 3 of granular retrosplenial cortex (Fig. 6*D*) (Kinnavane et al., 2018). Furthermore, the absence of anterograde label in the anterodorsal thalamic nuclei and the lateral mammillary bodies (Fig. 6*A*,*C*) demonstrates that the injections into the dorsal subiculum largely avoided uptake in the adjacent postsubiculum (van Groen and Wyss, 1990b; Wright et al., 2010). Moreover, there was no evidence of retrograde label in any of these sites. For example, the dorsal subiculum is reciprocally connected with the lateral entorhinal cortex (Witter et al., 1990): anterograde label was present in deep layers of the lateral entorhinal cortex, as expected based on the known topography of dorsal subicular terminations (Fig. 6*E*). At the same time, the connections from the lateral entorhinal cortex back to the dorsal subiculum arise in the more superficial layers (Witter and Amaral, 1991; Naber et al., 2001), but there was no evidence of retrograde label in these layers (Fig. 6*E*).

One DorSub_IDRDs animal was excluded because of only unilateral viral expression. Final group numbers were DorSub_iDRD = 11 and DorSub_Control = 6.

Before DREADD activation, both groups readily acquired the T-maze alternation task, and there were no differences in performance between the groups before the test sessions (*F*_(1,15)_ = 0.28, *p* = 0.60). Next, DREADD-mediated disruption of the dorsal subiculum did not appear to impair performance of the standard alternation task (i.e., when all task cues were available) (Fig. 5*C*). ANOVA yielded no effect of drug (*F*_(1,15)_ = 1.0, *p* = 0.33, η_p_^2^ = 0.063), group (*F*_(1,15)_ = 1.9, *p* = 0.19, η_p_^2^ = 0.112), or an interaction between these factors (*F*_(1,15)_ = 0.04, *p* = 0.84, η_p_^2^ = 0.003). This was confirmed by a Bayesian ANOVA. The model based on the interaction revealed a B_01_ factor of 4.114, indicating moderate evidence in favor of the null hypothesis.

In contrast, when task performance was more reliant on allocentric cues (maze rotation), DREADD-induced disruption of the dorsal subiculum impaired spatial working memory (Fig. 5*C*). There was an effect of drug (*F*_(1,15)_ = 9.9, *p* = 0.007, η_p_^2^ = 0.398), no effect of group (*F*_(1,15)_ = 0.55, *p* = 0.47, η_p_^2^ = 0.036), but importantly, a group × drug interaction (*F*_(1,15)_ = 14.2, *p* = 0.002, η_p_^2^ = 0.487). While there was no effect of clozapine treatment in the control group (*F*_(1,15)_ = 0.29, *p* = 0.61), clozapine selectively disrupted performance in the DorSub_iDRD group (*F*_(1,15)_ = 27.2, *p* = 0.001). Furthermore, performance under clozapine differed between the DREADD and control groups (*F*_(1,15)_ = 5.9, *p* = 0.028). Although impaired, the performance of the DorSub_iDRD group was above chance levels (*t*_(10)_ = 3.4, *p* = 0.007). Additional focused analysis assessed whether there was a difference in performance across the two versions of the tasks in the DorSub_iDRD group (under clozapine). This analysis confirmed that performance on the modified version was impaired relative to the standard task (*F*_(1,10)_ = 5.2 *p* = 0.046,η_p_^2^ = 0.343).

### Experiment 3: selective inhibition of ATN outputs to the subiculum and parahippocampal region impairs spatial working memory

**Figure 7.**
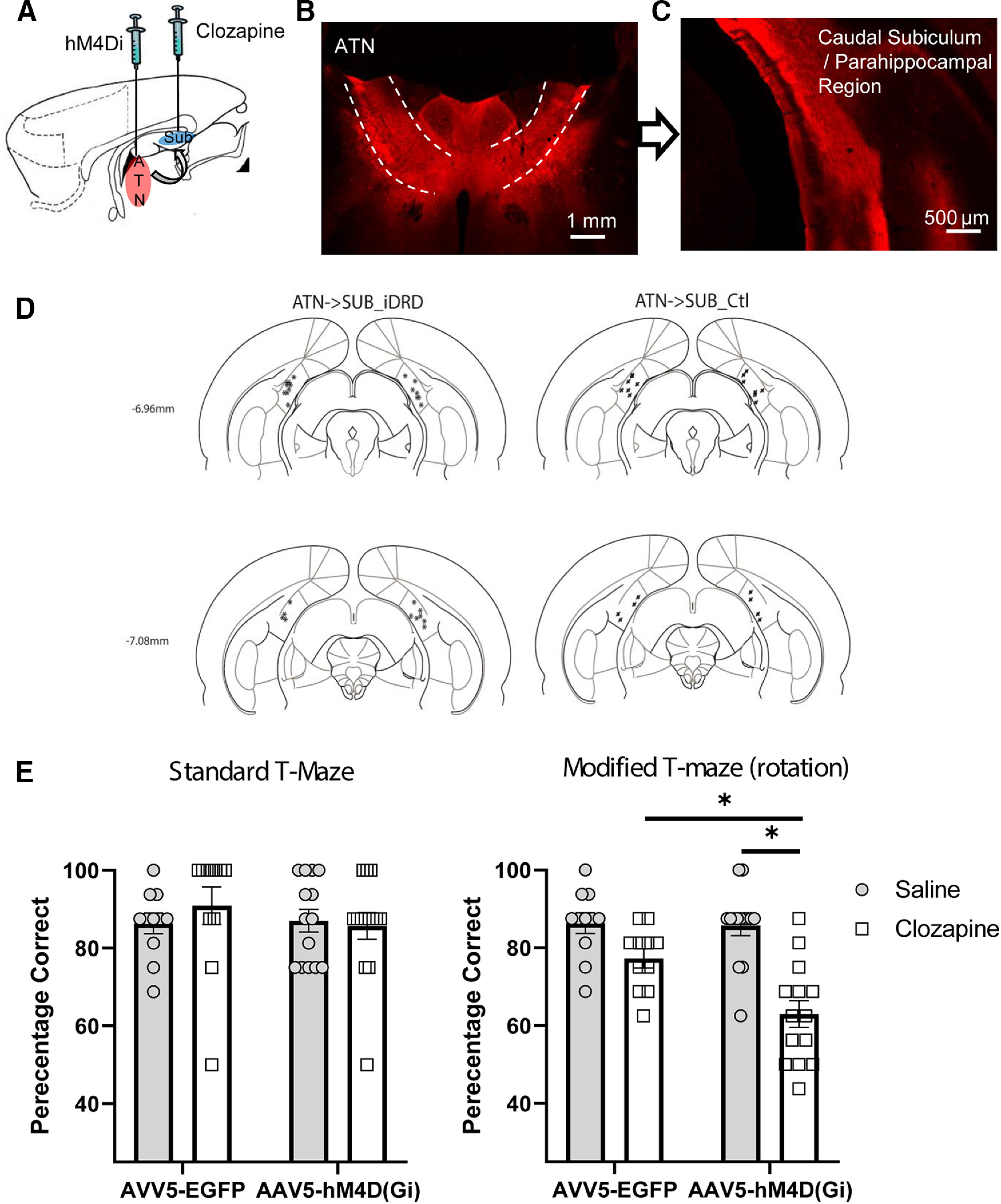
DREADD-mediated inhibition of anterior thalamic efferents to the subiculum and parahippocampal region impair spatial working memory. ***A***, Sagittal schematic of rat forebrain. ***B***, AAV5-hM4D(Gi)-mCherry expression in ATN (coronal section). All animals displayed robust expression within the ATN nuclei as well as (***C***) anterograde label in the caudal subiculum/parahippocampal region (right hemisphere; see also Fig. 4). ***D***, Diagrammatic coronal reconstructions showing the locations of cannulae aimed at the caudal subiculum/parahippocampal region in the experimental group (ATN→SUB_iDRD) and the control group (ATN→SUB_Ctl). Numbers indicate the distance (mm) from bregma (adapted from Paxinos and Watson, 2005). The tips of the injector cannulae extend 0.5 mm beyond the end of the guide cannulae. ***E***, DREADD activation did not disrupt spatial working memory on the standard version of the alternation task, but performance was impaired on the modified version (maze rotation; Fig. 2). The DREADD group was selectively impaired relative to their performance under saline (*p* < 0.001) as well as to the performance of controls under clozapine (*p* < 0.01). Plots represent mean percentage correct. Error bars indicate SEM. *denotes statistically significant differences.

Having established that chemogenetic disruption of the ATN (Experiment 1) or dorsal subiculum (Experiment 2) impairs distal cue-based spatial working memory, the next step was to target selectively the direct connections between these two sites. We therefore combined the axonal transport of the adeno-associated virus from the ATN (as in Experiment 1) with localized infusions of the DREADD ligand into the subiculum and parahippocampal region to disrupt inputs from the anterodorsal and anteroventral thalamic nuclei (Figs. 1, 7*A*).

Three ATN→Sub_iDRD animals were excluded as there was only very sparse label in the ATN. A further ATN→Sub_iDRD animal was excluded as the cannula blocked during the critical test sessions. The remaining animals displayed robust viral expression in the ATN as well as the expected profile of efferent label in the caudal subiculum/parahippocampal region (Fig. 7*B*,*C*) and other efferent targets (Fig. 5). Importantly, the pattern of terminal label within the parahippocampal sites and subiculum was consistent with known patterns of ATN termination within these sites (Fig. 5*A*). Furthermore, there was no evidence of retrograde label in the sites targeted with clozapine, the DREADD ligand (Fig. 5*A*). In 5 ATN→Sub_iDRD cases, there was also evidence of viral expression in the rostral ventral midline thalamic nuclei (reuniens and rhomboid nuclei). The cannula placements are shown in Figure 7*D*, noting that the actual site of infusion is a little ventral to that indicated. The final group numbers were ATN→Sub_iDRD = 14 and ATN→Sub _Control = 11.

Before DREADD activation, there were no differences between the two groups during pretest training on the T-maze task (*F*_(1,23)_ = 1.69, *p* = 0.21). Following DREADD activation, disconnection of the ATN projections to the caudal subiculum was without apparent effect on the standard spatial working memory task (i.e., when all cue types were available) (Fig. 2*A*). ANOVA yielded no effect of drug, group, or interaction between these factors (max *F*_(1,23)_ 0.681, *p* = 0.418, max η_p_^2^ = 0.029). A Bayesian ANOVA was also calculated, and the model based on the interaction revealed a B_01_ factor of 17.387, indicating strong evidence in favor of the null hypothesis.

However, when the task was modified by maze rotation (Fig. 2*B*), targeted disruption of the ATN efferents to the subiculum and parahippocampal region impaired T-maze alternation (Fig. 7*E*). There was an effect of infusion (*F*_(1,23)_ = 24.2, *p* = 0.00006, η_p_^2^ = 0.513) and of group (*F*_(1,23)_ = 6.6, *p* = 0.017, η_p_^2^ = 0.222), but also significantly an interaction between these factors (*F*_(1,23)_ = 4.5, *p* = 0.046 η_p_^2^ = 0.163). Simple main effects analysis confirmed that, in the ATN→SUB_iDRD group, the clozapine infusion impaired T-maze alternation behavior relative to performance after the saline infusion (*F*_(1,23)_ = 20.6, *p* ;= 0.00,015), but there was no statistical evidence for an effect of infusion in the control group (*F*_(1,23)_ = 3.5, *p* = 0.074). Importantly, performance following the clozapine infusion differed by group (*F*_(1,23)_ = 12.0, *p* = 0.0021) but not after the saline infusion (*F*_(1,23)_ = 0.025, *p* = 0.88). Additional analysis confirmed that, despite the DREADD-induced disruption of T-maze alternation, performance in the ATN→SUB_iDRD group remained above chance levels (*t*_(13)_ = 3.7, *p* = 0.002). Further focused analysis comparing the performance of the ATN→Sub_iDRD group (DREADDs activated) across the two variants of the T-maze task revealed an effect of task, as performance was significantly impaired following maze rotation relative to performance on the standard task (*F*_(1,13)_ = 25.9, *p* = 0.002, η_p_^2^ = 0.67).

Separate analyses comparing the performance the DREADD animals with or without additional DREADD expression in the ventral midline thalamus found no differences between these animals on the rotation trials when the DREADDs were activated (*F*_(1,23)_ = 0.009, *p* = 0.927). Unpublished data indicate that activation of the same DREADD construct by systemic clozapine (2 mg/kg) in the ventral midline thalamic nuclei is without apparent effect on either the standard or modified version of theT-maze tasks used here.

### Experiment 4: dorsal subiculum outputs to the ATN are vital for spatial working memory

**Figure 8.**
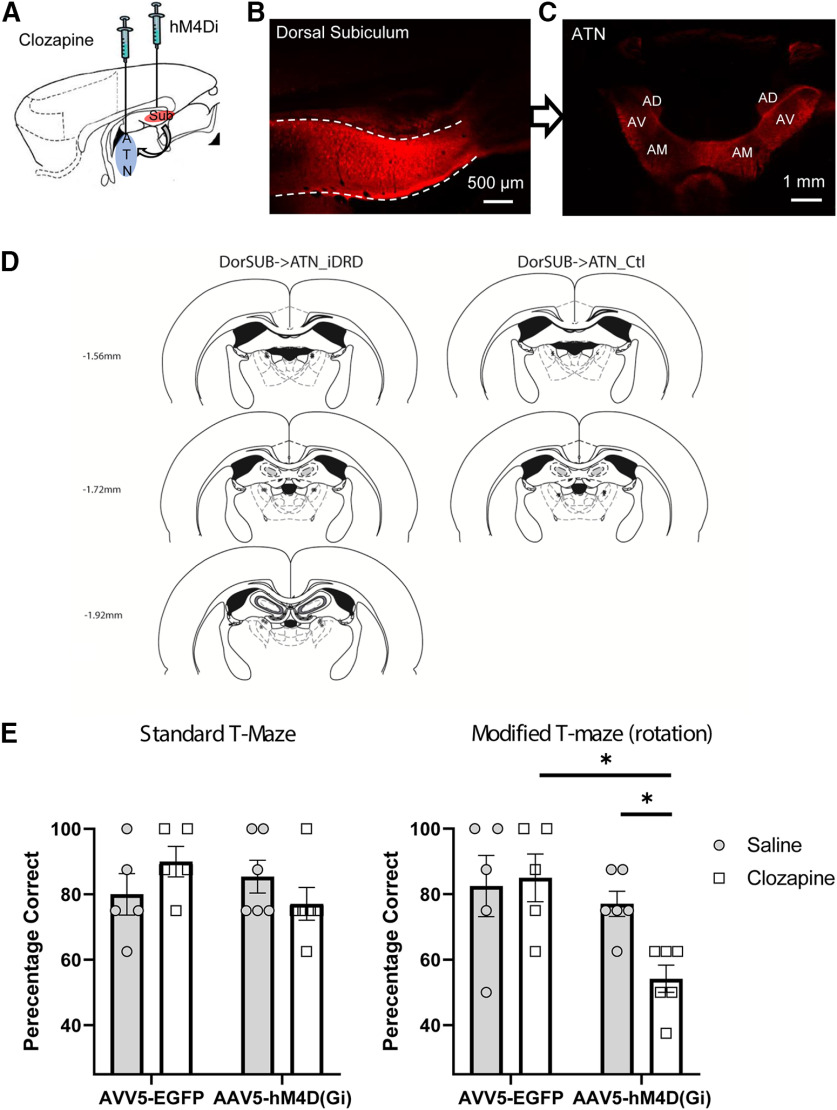
DREADD-mediated inhibition of dorsal subiculum efferents to the ATN impairs spatial working memory. ***A***, Sagittal schematic of rat forebrain. ***B***, AAV5-hM4D(Gi)-mCherry expression in dorsal subiculum (coronal section, left hemisphere). All animals displayed robust expression within the dorsal subiculum as well as (***C***) anterograde label in the ATN (see also Fig. 6). ***D***, Diagrammatic coronal reconstructions showing the locations of cannulae aimed at the ATN from the experimental group (ATN→SUB_iDRD) and the control group (ATN→SUB_Ctl). Numbers indicate the distance (mm) from bregma (adapted from Paxinos and Watson, 2005). The tips of the injector cannulae extend 0.5 mm beyond the end of the guide cannulae. ***E***, DREADD activation did not reliably disrupt spatial working memory on the standard version of the alternation task, but performance was impaired on the modified version (maze rotation; Fig. 2). The DREADD group was selectively impaired relative to their performance under saline (*p* < 0.01) as well as to the performance of controls under clozapine (*p* < 0.05). Plots represent mean percentage correct. Error bars indicate SEM. AD, anterodorsal; AM, anteromedial nucleus; AV, anteroventral nucleus. *denotes statistically significant differences.

In the final experiment, the importance of the direct connections from the dorsal subiculum to the ATN was tested. To this end, we combined injections of adeno-associated virus carrying the hMD4Di receptor into the dorsal subiculum with cannulae targeting dorsal subicular terminations in the ATN for subsequent infusion of the DREADD ligand (Fig. 8*A*).

All animals showed robust viral expression within the dorsal subiculum as well as anterograde transport to the ATN **(**Fig. 8*B*,*C*), with the densest label in the anteroventral and anteromedial thalamic nuclei, contrasting with much lighter label in the anterodorsal nucleus (Fig. 6*A*,*B*) as expected based on the known profile of dorsal subicular terminations within the ATN (Fig. 1) (Wright et al., 2010; Christiansen et al., 2016). Importantly, there was no evidence of retrograde label within the ANT (Fig. 6*A*,*B*). Within the mammillary bodies, anterograde viral expression was restricted to the medial mammillary nuclei (Fig. 6*C*), indicating that viral uptake did not extend to the postsubiculum (Allen and Hopkins, 1989; Wright et al., 2010). No animal was excluded on the basis of histologic assessment. However, one DorSub→ATN_iDRD rat was excluded as its cannulae blocked during the critical test sessions. The final group numbers were DorSub→ATN_iDRD = 6 and DorSub→ATN_Control = 5. Figure 8*D* shows the location of cannula placements within the ATN, noting that the actual site of infusion is a little ventral to that indicated.

There was no difference in T-maze alternation behavior between the two groups before DREADD activation (*F*_(1,9)_ = 2.0, *p* = 0.2). Likewise, disrupting dorsal subicular efferents to the ATN was without evident effect on the standard T-maze task (Fig. 8*E*). ANOVA revealed no effect of infusion, group, or interaction between these factors (max *F*_(1,9)_ = 2.9, *p* = 0.12, η_p_^2^ = 0.244). However, a Bayesian ANOVA was also calculated, and the model based on the interaction revealed a B_01_ factor of 2.926, indicating only anecdotal evidence in favor of the null hypothesis.

When the task was modified (maze rotation), targeted disruption of dorsal subicular inputs to the ATN led to a profound T-maze alternation deficit (Fig. 8*E*). ANOVA revealed no overall effect of infusion (*F*_(1,9)_ = 4.1, *p* = 0.067, η_p_^2^ = 0.318), but an effect of group (*F*_(1,9)_ = 6.3, *p* = 0.033, η_p_^2^ = 0.412) as well as an infusion × group interaction (*F*_(1,9)_ = 6.5, *p* = 0.031, η_p_^2^ = 0.419). Subsequent simple main effects analysis confirmed that there was no effect of infusion in the control group, but the clozapine infusion selectively disrupted T-maze performance in the DREADD group (*F*_(1,9)_ = 11.6, *p* = 0.0078). Further analysis revealed that there was no difference between the groups following the control infusions (*F*_(1,9)_ = 0.12, *p* = 0.75), but clozapine infusion impaired performance in the DorSub→ATN_iDRD group relative to the control group (*F*_(1,9)_ = 9.1, *p* = 0.014). Moreover, the T-maze alternation deficit in the DorSub→ATN_iDRD group was profound, as performance in these animals did not differ from chance levels (*t*_(5)_ =1, *p* = 0.368). Further focused analysis in the DSub→ATN_iDRD group (DREADDs activated) revealed an effect of task variant, as performance was significantly impaired following maze rotation relative to performance on the standard task (*F*_(1,5)_ = 20.9, *p* = 0.006, η_p_^2^ = 0.81).

## Discussion

Although it has long been appreciated that the hippocampal formation is functionally reliant on multiple brain sites, identifying those interactions most critical for memory remains an outstanding problem. The present study examined the ATN, which are strongly interconnected with the dorsal hippocampal formation, in particular, the dorsal subiculum (Meibach and Siegel, 1977; Shibata, 1993a; Christiansen et al., 2016). The two initial experiments confirmed the importance of the ATN and then the dorsal subiculum for spatial working memory by transiently inhibiting their respective activity. While the results partly mirror the effects of lesions in these two sites on reinforced spatial alternation (Means et al., 1971; Aggleton et al., 1995; Bannerman et al., 1999; McHugh et al., 2008; Aggleton and Nelson, 2015), they also appeared to differ in that the disruptive effects were more restricted to the maze rotation condition.

Next, the contributions of just the anterior thalamic–dorsal hippocampal region interconnections were isolated by separately targeting each pathway, first in one direction, then the other (Experiments 3, 4). These experiments not only revealed the importance of both sets of direct connections but also highlighted the significance of the dorsal subiculum (Experiment 4). Once again, spatial deficits most clearly emerged following maze rotation.

In all four experiments, spatial working memory was assessed using both a standard version of the alternation task as well as a more challenging “rotation” version. The latter version nullifies intramaze cues, which rats can readily use to assist spatial alternation (Douglas, 1966; Still and Macmillan, 1975; Dudchenko, 2001; Futter and Aggleton, 2006). The remaining strategies available to the animals on the modified version of the task consist of using the spatial disposition of distal cues (allocentric), as well as directional cues (Douglas, 1966; Dudchenko, 2001; Futter and Aggleton, 2006). Rats are, however, very poor at using egocentric (body turn), response, or path integration cues on the discrete trial version of the alternation task used in the current experiments, in which they are picked up and moved between sample and test (Baird et al., 2004; Futter and Aggleton, 2006). In contrast, the continuous version of the task used elsewhere may allow animals to adopt a response or egocentric strategy. The repeated ability of the experimental groups to solve the standard task presumably reflects their flexible reliance on intramaze cues and any remaining spatial information still capable of being processed.

Our finding that transiently disrupting the ATN (Experiment 1) can impair T-maze alternation consolidates evidence from lesion studies showing the importance of these nuclei for spatial working memory (Aggleton et al., 1996, 2009; Byatt and Dalrymple-Alford, 1996; Alcaraz et al., 2016; Perry et al., 2018). The current deficit was, however, arguably more nuanced with some evidence of an effect of DREADDs within the ATN on the standard version of the task, combined with a clearer deficit following maze rotation. Indeed, performance across the two task versions did not differ in this group, in contrast to the findings from Experiments 2-4. While this may reflect the higher dose of clozapine used in Experiment 1, it is broadly consistent with findings from lesion studies. Typically, permanent ATN lesions impair the standard version of the task with performance often not above chance levels (Aggleton and Nelson, 2015). The more selective deficit here may reflect how DREADD activation does not completely suppress neuronal activity within the target region (Smith et al., 2016). Another contributing factor may be that permanent ATN lesions cause appreciable dysfunctions in an array of distal sites, including the retrosplenial cortex and hippocampus, which may accentuate any behavioral effects (Jenkins et al., 2002; Poirier et al., 2008; Garden et al., 2009; Dupire et al., 2013).

DREADD-mediated disruption of the dorsal subiculum (Experiment 2) proved sufficient to impair spatial working memory, building on studies contrasting lesions in the dorsal and ventral hippocampal formation (Moser et al., 1993; Bannerman et al., 1999; McHugh et al., 2008; Strange et al., 2014). In those studies, selective dorsal hippocampal lesions impair reinforced alternation, whereas lesions of the ventral hippocampal formation appear to have little effect (Hock and Bunsey, 1998; Bannerman et al., 1999). The present DREADDs deficit was, however, only observed during maze rotation trials, where performance still remained above chance. This more focal effect accords with evidence from lesion studies that describe greater subiculum-related impairments on working memory tasks relative to reference memory (Galani et al., 1998; Potvin et al., 2007), alongside evidence that the dorsal subiculum supports the pattern separation of overlapping distal cues (Potvin et al., 2009), a process seen as integral to allocentric processing. Subiculum lesions also cause moderate Morris water maze deficits, again consistent with an incomplete allocentric deficit (Morris et al., 1990).

Disruption of the anterior thalamic projections to the subiculum and parahippocampal region (Experiment 3) impaired alternation following maze rotation. However, anterior thalamic projections not only reach the caudal subiculum but also innervate adjacent parahippocampal sites, including the postsubiculum, presubiculum, and parasubiculum (Shibata, 1993a). Furthermore, the cannulae placements indicate that the ligand infusions would have included the postsubiculum, as well as the caudal subiculum. Given the importance of the postsubiculum for head-direction information (Taube, 2007), any account of the deficit in Experiment 3 should incorporate a loss of parahippocampal head-direction information, which principally originates in the anterodorsal thalamic nucleus (Taube, 1995; Goodridge and Taube, 1997). At the same time, any potential involvement from the adjacent mediodorsal thalamic nucleus can be discounted in this experiment (and Experiment 4) as the mediodorsal nucleus is not directly connected with the dorsal subiculum, postsubiculum, or presubiculum (van Groen and Wyss, 1990a,b).

Consistent with the involvement of the anterodorsal thalamic-postsubiculum head direction signal is evidence that lesions of the postsubiculum produce mild deficits on both the radial-arm maze task, a test of spatial working memory, as well as the Morris water maze, a test of allocentric processing (Taube et al., 1992). Postsubiculum lesions also mildly impair directional responding in a water T-maze task (Peckford et al., 2014). One should, however, be cautious in concluding that the alternation deficits in Experiment 3 solely reflect the loss of head-direction information (Dillingham and Vann, 2019). The anteroventral thalamic nucleus projects to the dorsal hippocampal formation (Shibata, 1993a; Prasad and Chudasama, 2013), including the caudal subiculum. Moreover, lesions centered on the anteroventral thalamic nucleus are sufficient to impair spatial working memory (Aggleton et al., 1996; Byatt and Dalrymple-Alford, 1996). Furthermore, anteroventral nucleus neurons possess both theta and head-direction properties (Vertes et al., 2001; Albo et al., 2003; Tsanov et al., 2011), which likely contribute to thecomplex firing properties of neurons in the hippocampal formation that, in turn, support spatial and mnemonic processes (Jankowski et al., 2013).

The final experiment revealed that disrupting hippocampal efferents to the ATN impairs spatial alternation. This experiment is notable for several reasons. First, the effects can principally be ascribed to the dorsal subiculum efferents to the ATN. Consequently, the deficit is unlikely to reflect a loss of head-direction signaling. Visualization of axonal transport showed that it filled much of the anteroventral and anteromedial thalamic nuclei, with only very light signal in the anterodorsal nucleus (Fig. 6*A*,*B*). Likewise, the medial mammillary bodies contained considerable transported virus while the lateral mammillary nucleus lacked signal. These patterns of transport are consistent with uptake in the dorsal subiculum, with little or no postsubiculum or presubiculum involvement (van Groen and Wyss, 1990a,b).

A further feature of Experiment 4 was the severity of the spatial alternation deficit. Performance on the maze rotation condition was not above chance levels. This novel evidence for the importance of the direct projections from the hippocampal formation to the ATN complements the finding that lesions of the descending postcommissural fornix, which disconnect hippocampal inputs to the mammillary bodies, produce only mild behavioral deficits on tests of spatial memory, including spatial alternation (Vann et al., 2011; Vann, 2013). A clear implication is that the direct subicular projections to the ATN can effectively support spatial learning, even in the absence of hippocampal inputs to the mammillary bodies.

A striking feature of the current study concerns the consistency of the findings across the four experiments; that is, disrupting information flow from the hippocampal formation to the ATN and vice versa produced apparently comparable behavioral consequences. Nevertheless, the particular spatial learning processes disrupted by these various manipulations are most likely to be different, despite the similar phenotype. That the severest deficit occurred when subiculum efferents to the ATN were inhibited is, therefore, notable as this would largely spare head-direction information, pointing to an important involvement in allocentric processing.

Together, these findings inform our understanding of brain networks supporting memory. While pathway models of episodic memory have often emphasized the indirect hippocampal projections to the ATN via the mammillary bodies (Delay and Brion, 1969; Aggleton and Brown, 1999; Carlesimo et al., 2011; Bastin et al., 2019), the present study reveals the importance of the direct projections. Moreover, systems models of thalamic spatial processing have, understandably, largely focused on the head-direction pathway from the anterodorsal thalamic nucleus to hippocampal and parahippocampal regions (Goodridge and Taube, 1997; Taube, 2007; Winter et al., 2015). The current results not only reveal the functional significance of information flow from the hippocampus to the ATN but also highlight the key contributions of the anteromedial and anteroventral thalamic nuclei for spatial processing. This represents a significant realignment. More broadly, the results reveal functional interdependencies between two brain regions, helping to explain why pathology in either the medial diencephalon or the medial temporal lobes can result in profound anterograde amnesic syndromes with very similar core patterns of memory loss.
